# In the June 2010 issue

**DOI:** 10.1590/S1980-57642010DN40200001

**Published:** 2010

**Authors:** Ricardo Nitrini

**Affiliations:** Editor-in-Chief

In this issue of the journal we have published one systematic review, nine original articles and one case report with a review.

The review by **Anhoque et al**. revealed that cognitive impairment is present in clinically isolated syndrome and that its pattern is similar to that observed in patients with multiple sclerosis. This is a somewhat unexpected finding but follows the trend of other studies showing that cognitive disturbances are more frequent in many neurological disorders than formerly recognized.

Fonseca et al. investigated the occurrence of differences in episodic memory, concentrated attention and processing speed among four age groups of 136 neurologically healthy adults aged from 19 to 89 years. In general, differences were observed principally between young adults and elderly adults, but further studies are necessary to better analyze the performance of the middle-aged group in which differences on the delayed recall test are apparent from the age of 40 on.

The attitudes and knowledge of physicians on legal aspects regarding the autonomy of patients with Alzheimer’s disease were evaluated by a multidisciplinary group in the article by **Pioltini et al**. Interviews with physicians of different specialties revealed that half of interviewees had insufficient information on the legal aspects of caring for patients with Alzheimer’s disease. The authors concluded that there is a need to incorporate information on the legal aspects into professional training to improve the long-term management of patients with dementia.

**Cera et al.** investigated the types and frequencies of speech errors of Brazilian-Portuguese speakers with apraxia of speech. They evaluated 20 patients with apraxia of speech caused by stroke and found omissions to be one of the most frequent type of error, whereas addition errors were infrequent. These findings were different from those reported in English speaking patients, and probably are related to: differences in the methodologies for the classification of error types; the inclusion of speakers with apraxia secondary to aphasia; and the difference in the structure of Portuguese language to English.

**Park et al.** investigated the correlation between two neuroimaging features used for diagnosing Alzheimer’s disease: hippocampal volume and metabolic changes detected by proton spectroscopy. In a sample of 22 patients and 14 controls, proton spectroscopy of the posterior cingulated was the best predictor of the diagnosis of Alzheimer’s disease whereas no correlation was found between hippocampal volumes and spectroscopy in patients with Alzheimer’s disease.

**Lima-Silva et al.** evaluated the efficacy of a cognitive training program for memory and metamemory in a sample of relatively low educated older adults. The benefit of this intervention had been previously reported for older adults with ahigh education level. The five-session cognitive training was able to improve episodic memory and memory self-efficacy, an aspect of metamemory, in low educated individuals.

**Brucki et al.** analyzed the performance of low educated individuals from different social and cultural backgrounds on the Mini-Mental State Examination (MMSE). Subjects residing along the river banks of the Amazon region were matched by age and education to subjects living in Brazil’s largest city. There were differences on total scores of MMSE, with the subjects living in the urban region scoring higher than those living in the rural environment. Factors other than education influence results on screening tests.

**Porto et al.** analyzed the influence of educational level on performance on the Mattis Dementia Rating Scale, a neuropsychological battery frequently used for the diagnosis of dementia. Education had a significant influence on the total score and on scores of the subscales attention, initiation/perseveration and conceptualization. These findings highlight the importance of taking into account the effects of education on performance.

**Tedrus et al.** evaluated the impact of motor and non-motor symptoms on the quality of life in 20 patients with Parkinson’s disease. Besides motor symptoms, other symptoms such as language and attention impairments, as well as higher scores in the Hamilton Depression Rating Scale, were associated with worse quality of life in patients with Parkinson’s disease.

**Ferretti et al.** evaluated the accuracy of the diagnosis of dementia by the Brazilian Brain Bank of the Aging Brain Study Group, which relies solely on informant interview. The authors compared the sensitivity and specificity of the diagnosis based on informant interview against the diagnosis established at a memory clinic, considered the gold standard. They concluded that the informant interview has high specificity and sensitivity for the diagnosis of dementia as well as high specificity for the diagnosis of normal cognition.

**Siqueira et al.** reported a case of a 74-year-old woman with Alzheimer’s disease and chronic facial pain that had a significant improvement in cognition, functional activities and depressive symptoms after successful treatment of her facial pain. Given their review of the pertinent literature showed that patients with Alzheimer’s disease have higher compromise of oral health, with infections and teeth loss, investigation of orofacial pain should be carried out in all patients with Alzheimer’s disease.

**Ricardo Nitrini** Editor-in-Chief

